# A comprehensive multiplex PCR based exome-sequencing assay for rapid bloodspot confirmation of inborn errors of metabolism

**DOI:** 10.1186/s12881-018-0731-5

**Published:** 2019-01-06

**Authors:** Wenjie Wang, Jianping Yang, Jinjie Xue, Wenjuan Mu, Xiaogang Zhang, Wang Wu, Mengnan Xu, Yuyan Gong, Yiqian Liu, Yu Zhang, Xiaobing Xie, Weiyue Gu, Jigeng Bai, David S. Cram

**Affiliations:** 1 Children and Women’s Hospital of Shanxi, Women Health Center of Shanxi, Taiyuan, Shanxi China; 2 Children and Women’s Hospital of Shanxi, Newborn Disease Screening Center of Shanxi Province, Taiyuan, Shanxi China; 3 Beijing Berry Genomics Corporation, Building 5, Courtyard 4, Yiliaoyuan Road, ZGC Life Science Park, Beijing, 102206 Changping District China; 4 Beijing Chigene Translational Medicial Research Center Co., Beijing, 101111 China

**Keywords:** Newborns, Inborn errors of metabolism (IEM), Compound heterozygotes, PCR exome amplification and re-sequencing (PEARS), Phenylketonuria (PKU), Tandem mass spectroscopy (MS MS)

## Abstract

**Background:**

Tandem mass spectrometry (MS MS) and simple fluorometric assays are currently used in newborn screening programs to detect inborn errors of metabolism (IEM). The aim of the study was to evaluate the clinical utility of exome sequencing as a second tier screening method to assist clinical diagnosis of the newborn.

**Methods:**

A novel PCR-exome amplification and re-sequencing (PEARS) assay was designed and used to detect mutations in 122 genes associated with 101 IEM. Newborn bloodspots positive by biochemical testing were analysed by PEARS assay to detect pathogenic mutations relevant to the IEM.

**Results:**

In initial validation studies of genomic DNA samples, PEARS assay correctly detected 25 known mutations associated with 17 different IEM. Retrospective gene analysis of newborns with clinical phenylketonuria (PKU), identified compound heterozygote phenylalanine hydroxylase (*PAH)* gene mutations in eight of nine samples (89%). Prospective analysis of 211 bloodspots correctly identified the two true PKU samples, yielding positive and negative predictive values of 100%. Testing of 8 true positive MS MS samples correctly identified potentially pathogenic compound heterozygote genotypes in 2 cases of citrullinemia type 1 and one case each of methylmalonic acidemia, isobutyryl-CoA dehydrogenase deficiency, short chain acyl-CoA dehydrogenase deficiency and glutaric acid type II and heterozygous genotypes in 2 cases of autosomal dominant methioninemia. Analysis of 11 of 12 false positive MS MS samples for other IEM identified heterozygous carriers in 8 cases for the relevant genes associated with the suspected IEM. In the remaining 3 cases, the test revealed compound heterozygote mutations in other metabolic genes not associated with the suspected IEM, indicating a misinterpretation of the original MS MS data.

**Conclusions:**

The PEARS assay has clinical utility as a rapid and cost effective second-tier test to assist the clinician to accurately diagnose newborns with a suspected IEM.

**Electronic supplementary material:**

The online version of this article (10.1186/s12881-018-0731-5) contains supplementary material, which is available to authorized users.

## Background

Inborn errors of metabolism (IEM) are a heterogeneous group of genetic disorders caused by specific enzyme defects in complex metabolic pathways, resulting in a perturbed metabolic state due to either depletion of essential metabolites or the accumulation of abnormal levels of toxic metabolites. Individually each IEM is relatively rare in the general population, but collectively depending on ethnicity and geographical location, 1 in 1000–4000 newborns are affected with a metabolic disorder [1, 2]. Depending on the type of IEM and the severity of the pathogenic mutations, there are broad spectrums of clinical manifestations ranging from early neonatal death and mental retardation to mild metabolic defects that are controllable by manipulation of diet [2–4]. The vast majority of IEM are caused by autosomal recessive mutations in genes that encode catalytic enzymes responsible for amino acid, organic acid or fatty acid synthesis [5]. Genotype-phenotype studies of compound heterozygotes have shown that the clinical phenotype of each disease is usually dependent on the expression level of the allele harbouring the less severe pathogenic mutation [6, 7].

Phenylalanine hydroxylase (PAH) deficiency, traditionally known as phenylketonuria (PKU), is the most common IEM with a birth incidence of 1 in 2500 to 10,000 [8, 9]. PKU is characterised by high levels of blood phenylalanine from birth which results in a state of hyperphenylalaninemia, and if undiagnosed, can lead to severe neurological and neuropsychological symptoms, seizures, ataxia and psychosocial problems [10, 11]. Over 95% of hyperphenylalaninemia is caused by mutations in the *PAH* gene which is responsible for the conversion of phenylalanine to tyrosine [9]. Familial studies have identified more than 500 pathogenic DNA variants located randomly across coding and non-coding regions of the *PAH* gene, and novel mutations continue to be identified [6, 12]. A minority of patients with hyperphenylalaninemia (< 5%) have a deficiency in tetrahydrobiopterin (BH4) synthesis, an essential cofactor required for hydroxylation of phenylalanine [13]. BH4 deficiency is caused by autosomal recessive mutations in the genes *PTS* and *GCH1* encoding BH4 biosynthesis enzymes, and in genes *PCBD1* and *QDPR* encoding BH4 regeneration enzymes.

Over the last 50 years, newborn screening programs have been introduced globally into major hospitals for early detection of common IEM [14]. Throughout China, most newborn screening laboratories routinely screen bloodspots for PKU, congenital hypothyroidism (CH) and congenital adrenal hyperplasmia (CAH) using fluorometric assays, which are highly sensitive and specific for disease detection [15, 16]. In addition, many laboratories have also implemented tandem mass spectroscopy (MS MS) testing as a first tier cost effective screening methodology, which has the capacity to detect over 30 types of IEM [17–21]. More recently next generation sequencing (NGS) has been considered as an emerging tool to assist with the diagnosis of newborns with IEM [22]. To further explore the clinical utility of gene testing for detecting IEM, the aim of our study was to evaluate the performance of a novel NGS method for newborn bloodspot gene testing termed PCR Exome Amplification and Re-Sequencing (PEARS), which was designed specifically for detection of pathogenic mutations in 122 genes associated with 101 different IEM.

## Methods

### Study design and newborn samples

The clinical research study was approved by the Ethics Committee of the Children’s Hospital of Shanxi (IRB-XJS-2015-2). Couples provided written consent for collection, storage and analysis of bloodspot samples from their newborn children. Bloodspot samples tested included those from newborns diagnosed with PKU (*n* = 9), prospective newborns for PKU analysis (*n* = 211) and newborns positive by MS MS (*n* = 20), comprising 8 true positives and 12 false positives. In families where trio analysis was requested to confirm parental disease inheritance, parents also provide written informed consent for collection of their peripheral blood samples.

### Collection and processing of newborn bloodspot samples for testing

Peripheral blood (100 μl) samples from a heal prick were spotted onto Whatman FTA paper cards (Whatman) in triplicate, dried and cards stored in a desiccator. The first bloodspot was used for biochemical analysis of blood metabolite levels and a second bloodspot used to isolate genomic DNA (TIANamp Blood Spots DNA Kit, TIAGEN). For trio studies to confirm parental DNA variants found in the proband, genomic DNA samples were isolated from peripheral blood using a DNeasy Blood and Tissue kit (Qiagen).

### Biochemical metabolite assays

Bloodspot levels of free phenylalanine were measured using an ELISA based fluorometric assay (Neonatal Phenylalanine Kit, Labsystems Diagnostics Oy, China). Phenylalanine levels were expressed as milligrams per decilitre (mg/dL) of blood. Newborn PKU was classified as mild when phenylalanine levels were 2–6 mg/dl, moderate when levels were 6–20 mg/dl and severe when levels exceeded 20 mg/dl [23]. Tandem mass spectroscopy (MS MS) testing of blood spots was performed using the NeoBaseTM Non-derivatized MSMS kit (PerkinElmer, Jiangsu, China). IEM were called based on previously defined clinically validated cut-off target ranges for primary metabolites altered for each IEM [24].

### PCR exome amplification and re-sequencing for profiling mutations in metabolic genes

Development, optimization and validation of the PEARS-101 test, as well as the testing of clinical newborn bloodspots, was performed by the next generation sequencing (NGS) research laboratory at Berry Genomics, Beijing. The PEARS-101 test was designed to screen for mutations in 122 genes associated with 101 different metabolic disorders (Additional file 1: Table S1). The selected 101 metabolic disorders included the 45 disorders that are routinely tested by MS MS in our newborn screening program and 56 other common IEM that are not detectable by MS MS. To generate the metabolic gene panel, exonic and immediately adjacent intronic sequences of the 122 IEM genes were amplified by targeted multiplex PCR using to produce an exome library with a size range of 150–275 bp that is amenable to next generation sequencing (NGS). In the case of larger exonic regions, we designed primers to generate overlapping amplicons. Primer pairs were designed using Ion AmpliSeq Designer Software (Thermo Fisher). The final primer sets were divided into two separate multiplex PCR reactions, comprising multiplex 1 with 998 primer pairs (pool 1) and multiplex 2 with 984 primer pairs (pool 2).

A series of PCR optimisation experiments using DNA extracted from newborn bloodspots was performed by varying final primer concentrations (5–10 μM) and primer annealing temperature (55–60 °C). Poorly performing primers creating primer-dimers were redesigned and retested. The final optimised multiplex PCR reactions contained 2 μl DNA template (20 ng of bloodspot DNA), 10 μl 2X Platinum multiplex PCR Master mix (Applied Biosystems), 1 μl GC enhancer (Applied Biosystems), 2 μl of 100 μM pool 1 or pool 2 primer mix (forward and reverse primer pairs) and 5 μl sterile water to a final volume of 20 μl. The final multiplex PCR reaction was performed using the S1000 PCR machine (Bio-Rad): 1 cycle at 95 °C for 2 min, followed by 21 cycles at 95 °C for 30 s, 58 °C for 90 s and 72 °C for 60 s, then a final extension at 72 °C for 20 min.

Following PCR, multiplex 1 and 2 reaction products were pooled together, end repaired and 3′ end adenylated using a combination of T4 DNA polymerase, T4 PNK and Klenow Fragment (3′ → 5′ exo–) enzymes (Enzymatics). Amplicons were purified using 1.5X Ampure XP beads (Beckman) and then ligated with Illumina Truseq adapters including a unique 7 bp barcode for sample identification, followed by 0.7X Ampure beads purification to remove excess non-ligated adaptors. Exome libraries were then quantified by PCR performed on the StepOne Plus machine (Applied Biosystems) and then subjected to paired end sequencing (2 × 250 bp) on the HiSeq2500 platform (Illumina), to generate a minimum of 4 million paired end (PE) reads.

### QC assessment of PEARS-101 test

The optimised PEARS-101 test designed to target the exome of 101 metabolic disorders was evaluated for key quality control (QC) performance indicators by analyzing ten independent bloodspot samples. QC results were consistent across all ten bloodspots (Additional file 2: Figure S1, typical QC results). The IEM exome libraries generated from both multiplex PCR reactions P1 and P2 comprised DNA molecules ranging from 150 to 275 bp in size, and were consistent with the size of the final exome library tagged with 126 bp barcoded Illumina adaptors (Additional file 2: Figure S1A). Between 4 and 8 million 250 PE reads were sequenced per sample, with a sequencing depth (coverage) ranging between 300X and 7,000X (Additional file 2: Figure S1B). Over 90% of the exome sequences were sequenced at a depth of >500X. Further, based on the presence of heterozygous SNPs within the exomes, PCR allelic bias was measured as minimal, with the majority of SNPs showing an allelic ratio (AR) close to 1 (range 0.5 to 1.4) and a minor population of SNPs (outliers) with more biased allelic ratios of 0.35 and 1.7. Using this plot, we arbitrarily assigned ARs for calling heterozygotes (0.3 < AR < 1.75) and for calling mosaics (AR < 0.3 and > 1.7).

Based on these preliminary assessments, the optimised PEARS-101 gene assay passed the necessary requirements of a robust test for producing representative and relatively unbiased sequencing data from newborn bloodspot samples, allowing us to make confident calls of the newborn genotypes. In all analyses of newborn clinical bloodspot samples, QC analysis was incorporated into the test pipeline, to ensure that these QC standards were met. Further, QC results were attached to the final genetics report of the newborn as an Appendix.

### Analysis and confirmation of DNA variants

Reads were aligned to the human reference genome sequence (UCSC hg19) using Burrows-Wheeler Aligner (BWA) MEM. DNA variants were identified using Genome Analysis Toolkit (GATK version 7, https://software.broadinstitute.org/gatk), Atlas2 (version 1.4.2, https://www.hgsc.bcm.edu/software/atlas-2) and Platypus (version 0.8.1, http://www.well.ox.ac.uk/platypus). Annovar was used to annotate functional information for all DNA variants. Based on guidelines from the American College of Medical Genetics (ACMG) [25], variants were classified as pathogenic, likely pathogenic, variants of uncertain significance or likely benign. Standard Sanger sequencing of DNA samples from newborns or family trios was performed to confirm newborn genotypes.

### Genetic counseling

DNA variants detected in newborns that were classified as either pathogenic, likely pathogenic or of uncertain significance were reported to the clinician. In regard to variants of uncertain significance, we routinely searched available genetic databases (including chinese databases) to determine their population frequency and thus likelihood of being pathogenic. Variants classified as benign were not reported. Following discussion of their child’s genetic report with their clinician, parents were offered genetic counseling from the resident hospital clinical geneticist specializing in IEM to assess the risk and potential severity of the indicated IEM and discuss potential treatments. In cases where the child had not developed any clinical signs of the IEM, the clinical geneticist recommended regular monitoring of their child for any signs of the disease phenotype.

## Results

### Performance of the PEARS-101 test for detection of pathogenic mutations in metabolic disease genes

For initial validation of the PEARS-101 test, the newborn screening laboratory selected 8 peripheral blood genomic DNA samples harbouring known pathogenic variants that were previously identified by Sanger sequencing (Table 1). PEARS-101 analysis correctly identified all nine primary pathogenic variants in the eight samples and confirmed samples GD-1 and GD-2 as compound heterozygous for *PAH* pathogenic variants c.331C > T/c.442-1G > A and *PTS* mutations c.166G > A/c.286 G > A, respectively. In addition, PEARS-101 correctly identified a further two heterozygous *PAH* variants c.441 + 3G > C and c.728G > A (GD-3 and GD-4), one heterozygous *MUT*variant c.1741C > T (GD-5) and three heterozygous *ACADS* variants c.164C > T (GD-6) and c.66A > G (GD-7 and GD-8). Apart from these nine types of primary mutations, samples were also identified with other heterozygous variants in one or more secondary metabolic genes (Table 1). All nine primary mutations (Additional file 3: Figure S2) and all 17 newly detected secondary mutations (Additional file 4: Figure S3) were confirmed by Sanger sequencing. Based on these validations studies involving 17 of the 122 (16%) testable genes in the full panel, the PEARS-101 assay proved highly sensitive and specific for detection of 25 DNA variants associated with 17 different IEM.

**Table 1:** Validation of the PEARS-101 test using newborn genomic DNA samples with known IEM gene variants

Newborn	Detection by Sanger sequencing		Detection by PEARS-101 test	
	Gene variant	Predicted amino acid change	Primary variants	Secondary variants
GD-1	c.331C > T (*PAH*)	p.R111X	c.331C > T (*PAH*)	c.1562G > C (*ALDH4A1*) c.1001G > T (*ETFA*) c.814G > A (*PEX1*) c.367G > T (*PHYH*)
	c.442-1G > A (*PAH*)	No change	c.442-1G > A (*PAH*)	
GD-2	c.166G > A (*PTS*)	p.V56 M	c.166G > A (*PTS*)	c.1111delC (*HGD*)
	c.286G > A (*PTS*)	p.D96N	c.286G > A (*PTS*)	
GD-3	c.441 + 3G > C (*PAH*)	No change	c.441 + 3G > C (*PAH*)	c.1367G > A (*GLDC*)
GD-4	c.728G > A (*PAH*)	p.R243Q	c.728G > A (*PAH*)	c.496A > G (*DPYD*) c. 1027A > C (*HGD*) c.1574 T > C (*TSHR*)
GD-5	c.1741C > T (*MUT*)	p.R581X	c.1741C > T (*MUT*)	c.2194G > A (*DPYD*) c.443A > G (*HMGCL*) c.613A > G (*GALT*)
GD-6	c.164C > T (*ACADS*)	p.P55L	c.164C > T (*ACADS*)	None
GD-7	c.66A > G (*ACADS*)	p.W22X	c.66A > G (*ACADS*)	c.512G > A (*GRHPR*) c.158G > A (*PAH*) c.608 T > C (*SARDH*) c.1229G > A (*SLC22A5*)
GD-8	c.66A > G (*ACADS*)	p.W22X	c.66A > G (*ACADS*)	c.1229G > A (*SLC22A5*)

*GD* Genomic DNA. All mutations detected by PEARS-101 were confirmed by PCR Sanger sequencing (Additional file 3: Figure S2, Additional file 4: Figure S3)

### Gene analysis of bloodspots from newborns diagnosed with PKU

**Fig. 1:**
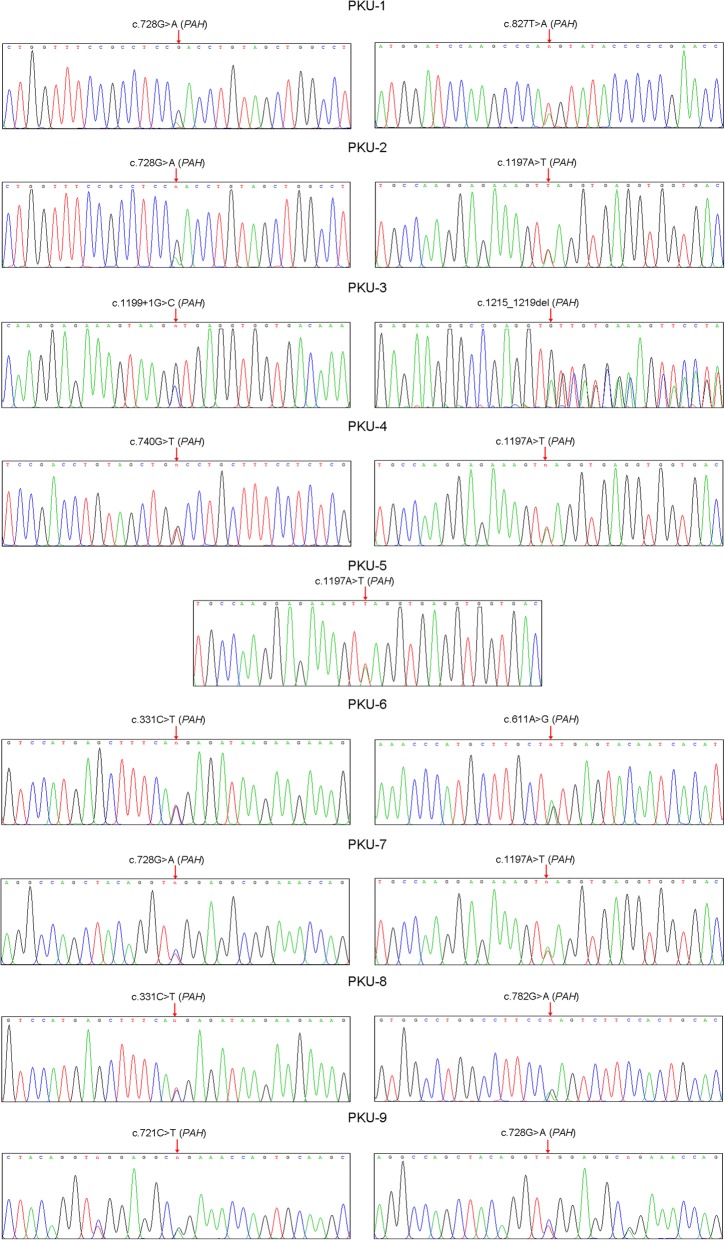
Confirmation of *PAH* pathogenic variants detected in newborns with PKU by Sanger sequencing

In order to evaluate the sensitivity of the PEARS-101 test for detecting genetic variants associated with PKU, nine bloodspot samples (PKU 1–9), positive from newborn primary screening and clinically diagnosed with PKU, were retrospectively tested (Table 2). PEARS-101 detected two pathogenic *PAH* variants in eight of the nine samples (89%). In the remaining sample PKU-5, only one pathogenic *PAH* variant c.1197A > T was detected, which is a silent variant that induces post-transcriptional skipping of exon 11 [26]. All *PAH* pathogenic variants associated with the eight compound heterozygotes and the one heterozygote were confirmed by Sanger sequencing (Fig. 1). Sample PKU-5 was referred for confirmatory whole exome sequencing (WES) using 2μg of peripheral blood genomic DNA. Analysis of the WES sequencing data confirmed the c.1197A > T variant, but failed to find a second *PAH* variant within the exon and intron boundary regions of the *PAH* gene. Thus, the PEARS-101 test correctly identified pathogenic genotypes for 8 of 9 newborns (89%) with clinical PKU.

**Table 2:** Comparison of biochemical and gene testing for diagnosis of PKU

Newborn	Fluorometric assay Phenylalanine levels (mg/dl)			PEARS-101 test			
	Original bloodspot	Repeat bloodspot	PKU Diagnosis	P*AH* variants detected	Predicted amino acid change	Pathogenicity Classification*	Diagnosis
Retrospective study (*n* = 9)							
PKU-1	9.71	10.5	Moderate	c.827 T > A	p.M276K	Pathogenic	PKU
				c.728G > A	p.R243Q	Pathogenic	
PKU-2	15.4	40.4	Severe	c.1197A > T	p.V399 V^#^	Pathogenic	PKU
				c.728G > A	p.R243Q	Pathogenic	
PKU-3	19.1	21.7	Severe	c.1215_1219del	p.T405 fs	Pathogenic	PKU
				c.1199 + 1G > C	mRNA splicing	Pathogenic	
PKU-4	26.9	36.2	Severe	c.1197A > T	p.V399 V^#^	Pathogenic	PKU
				c.740G > T	p.G247 V	Pathogenic	
PKU-5	14.6	45.8	Severe	c.1197A > T	p.V399 V^#^	Pathogenic	Normal
PKU-6	12.5	39.2	Severe	c.331C > T	p.R111X	Pathogenic	PKU
				c.611A > G	p.Y204C	Pathogenic	
PKU-7	22.8	30.0	Severe	c.1197A > T	p.V399 V^#^	Pathogenic	PKU
				c.728G > A	p.R243Q	Pathogenic	
PKU-8	19.3	30.9	Severe	c.331 C > T	p.R111X	Pathogenic	PKU
				c.782G > A	p.R261Q	Pathogenic	
PKU-9	6.3	9.7	Moderate	c.721C > T	p.R241C	Pathogenic	PKU
				c.728G > A	p.R243Q	Pathogenic	
Prospective study (*n* = 211)							
S-43	10.4	36.9	Severe	c.611A > G	p.Y204C	Pathogenic	PKU
				c.47-48del	p.S16 fs	Pathogenic	
S-109	5.4	8.1	Moderate	c.782G > A	p.R261Q	Pathogenic	PKU
				c.721C > T	p.R241C	Pathogenic	
S-119	< 2.0	ND	Normal	c.1064C > A	p.Y356X	Pathogenic	Normal
S1-S208*	< 2.0	ND	Normal	None	Not relevant	Not relevant	Normal

*By definition of a clinical diagnosis of PKU, all newly detected PKU variants can be classified as pathogenic; ^#^ Silent mutation; *ND* Not Done; *S1-S208 (excluding S-43, S-109 and S-119)

To extend these studies, we obtained a set of 211 prospective bloodspot samples assayed by the phenylalanine fluorometric assay whereby two bloodspots were reported with elevated levels of phenylalanine and the other 209 newborn blood spots were reported with normal levels of phenylalanine (Table 2). Positive newborn samples S-43 and S-109 were subsequently confirmed to have clinical PKU. The 211 reserve bloodspots were coded by the newborn screening laboratory and then sent for PEARS-101 testing. After decoding results, the two true PKU positive newborns samples S-43 and S-109 were identified with compound heterozygous *PAH* pathogenic variants c611A > G/c.47_48del and c.782G > A/c.721C > T, respectively. For the 209 true PKU negatives, newborn sample S-119 was identified as a heterozygote carrier of the pathogenic *PAH* variant c.1064C > A and the remaining 208 newborns did not carry any *PAH* variants or other variants in the *PTS*, *GCH1, PCBD1* and *QDPR* genes associated with BH4 deficiency. Taken together, based on this prospective data set, the PEARS-101 test had a positive predictive value (PPV) and a negative predictive value (NPV) of 100% for diagnosing PKU.

### Gene analysis of 20 MS MS positive newborn bloodspot samples

**Fig. 2:**
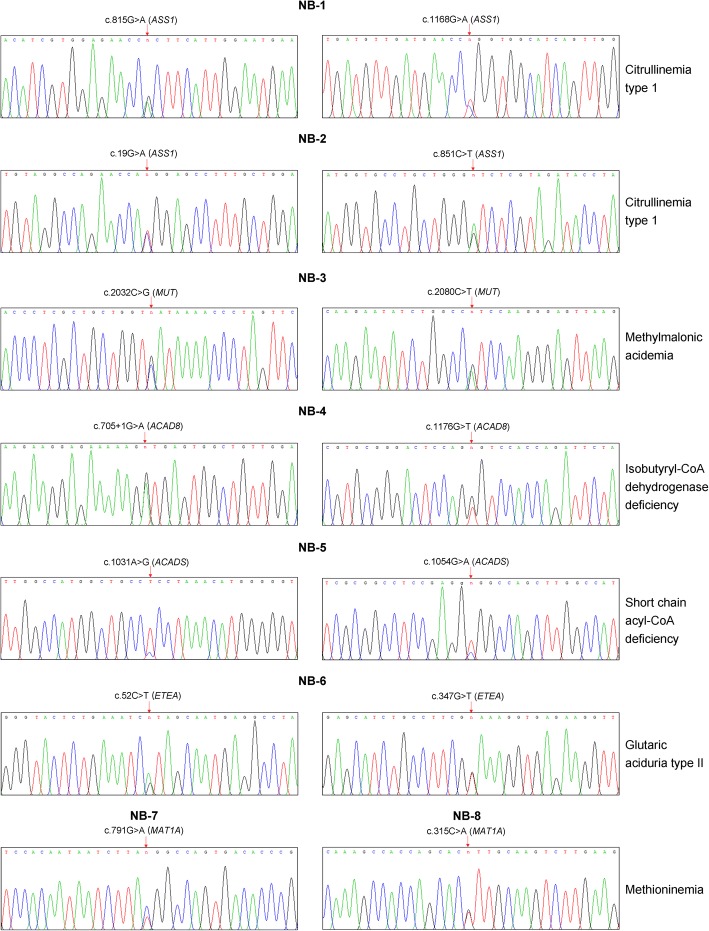
Sanger sequencing confirmation of pathogenic IEM variants detected in 8 newborns diagnosed with an IEM (true positives)

To assess the ability of the PEARS-101 test to accurately determine IEM genotypes, we retrospectively analysed 20 MS MS positive bloodspots samples where the laboratory had been informed of the suspected IEM diagnosis (Table 3). Based on repeat bloodspot testing by MS MS and specific biochemical testing for the indicated IEM, 8 newborns (NB 1–8) were confirmed as true positives and 12 newborns (NB 9–20) confirmed as false positives. The 8 true positive newborns included 2 cases of methioninemia, 2 cases of citrullinemia type 1, and one case each of methylmalonic acidemia, isobutyryl-CoA dehydrogenase deficiency, short chain acyl-CoA dehydrogenase deficiency and glutaric acid type II. In 6 of these 8 cases (excluding 2 cases of methioninemia), gene testing identified compound heterozygous pathogenic variants in the relevant genes associated with diagnosed IEM. In the 2 cases of methioninemia, which can be inherited in an autosomal dominant mode, a heterozygous pathogenic variant was identified in both cases. All mutations in NB 1–8 were subsequently confirmed by Sanger sequencing (Fig. 2).

**Table 3:** Comparison of MS MS and gene testing for diagnosis of IEM

MS MS positive bloodspot	MS MS test		Clinical diagnosis	PEARS-101 test				
	Primary metabolite changes	Suspected IEM		Gene	Relevant pathogenic variants detected	Predicted aa change	Pathogenicity Classification^a^	Indicated IEM status
True positives								
NB-1	Cit ↑	Citrullinemia type 1	Citrullinemia type 1	*ASS1*	c.815G > A	p.R272H	Pathogenic	Citrullinemia type 1
					c.1168G > A	p.G390R	Pathogenic	
NB-2	Cit ↑	Citrullinemia type 2	Citrullinemia type 2	*ASS1*	c.19G > A	p.V7M	Pathogenic	Citrullinemia type 2
					c.851C > T	p.T284I	Pathogenic	
NB-3	C3 ↑	Methylmalonic acidemia	Methylmalonic acidemia	*MUT*	c.2032C > G	p.H678D	Pathogenic	Methylmalonic academia
					c.2080C > T	p.R694W	Pathogenic	
NB-4	C4 ↑	Isobutyryl-CoA dehydrogenase deficiency	Isobutyryl-CoA dehydrogenase deficiency	*ACAD8*	c.705 + 1G > A	No change	Pathogenic	Isobutyryl-CoA dehydrogenase deficiency
					c.1176G > T	p.R392S	Pathogenic	
NB-5	C4 ↑ C4/C2 ↑	Short chain acyl-CoA dehydrogenase deficiency	Short chain acyl-CoA dehydrogenase deficiency	*ACADS*	c.1031A > G	p.E344G	Pathogenic	Short chain acyl-CoA dehydrogenase deficiency
					c.1054G > A	p.A352T	Pathogenic	
NB-6	C4↑, C5↑, C5DC↑, C6↑, C8↑, C10↑	Glutaric aciduria type II	Glutaric aciduria type II	*ETFA*	c.52C > T	p.R18X	Pathogenic	Glutaric aciduria type II
					c.347G > T	p.G116 V	Pathogenic	
NB-7	Met ↑	Methioninemia	Methioninemia	*MAT1A*	c.791G > A	p.R264H	Pathogenic	Methioninemia
NB-8	Met ↑	Methioninemia	Methioninemia	*MAT1A*	c.315C > A	p.N105K	Pathogenic	Methioninemia
False positives								
NB-9	Cit ↓	Ornithine transcarbamylase deficiency (male)	Normal	*OTC*	c.809A > G	p.Q270R	Uncertain	Male carrier of Ornithine transcarbamylase deficiency
					c.137A > G	p.K46R	Likely benign	
NB-10	Ala ↑	Hyperalaninemia	Normal	*SARDH*	c.2050A > G	p.S684G	Uncertain	Compound heterozygote for Sarcosinuria
					c.1738 T > C	p.Y580H	Uncertain	
NB-11	Leu ↑	Maple syrup urine disease	Normal	*MMACHC*	c.458G > A	p.R153Q	Uncertain	Compound heterozygote for Methylmalonic aciduria
					c.799C > T	p.R267W	Uncertain	
NB-12	Leu ↑	Maple syrup urine disease	Normal	*ETFDH*	c.1823G > A	p.G608D	Uncertain	Compound heterozygote for Glutaric aciduria type II
					c.770A > G	p.Y257C	Uncertain	
NB-13	C4 ↑	Short-chain acyl-CoA dehydrogenase deficiency	Normal	*ACADS*	c.1153G > A	p.A385T	Uncertain	Carrier of Short-chain acyl-CoA dehydrogenase deficiency
NB-14	Cit ↑	Citrullinemia type I	Normal	*ASS1*	c.910C > T	p.R304W	Uncertain	Carrier of Citrullinemia type I
NB-15	Cit ↑	Citrullinemia type 1	Normal	*ASS1*	c.1176_1178del	p.392_393del	Uncertain	Carrier of Citrullinemia type I
NB-16	C0 ↓	Primary carnitine deficiency	Normal	*SLC22A5*	c.1400C > G	p.S467C	Uncertain	Carrier of Primary carnitine deficiency
NB-17	C5OH ↑	3 Methylcrotonyl Coenzyme A dehydrogenase deficiency	Normal	*MCCC1*	c.328C > T	p.Q110X	Uncertain	Carrier of 3 Methylcrotonyl Coenzyme A dehydrogenase deficiency
NB-18	C4 ↑	Isobutyryl-CoA dehydrogenase deficiency	Normal	*ACAD8*	c.712delT	p.W238 fs	Uncertain	Carrier of Isobutyryl-CoA dehydrogenase deficiency
NB-19	C14:1 ↑ C14:2 ↑ C14 ↑	Very long-chain 3-OH acyl-CoA dehydrogenase deficiency	Normal	*ACADVL*	c.1748C > T	p.S583 L	Uncertain	Carrier of very long-chain 3-OH acyl-CoA dehydrogenase deficiency
NB-20	C0 ↓	Primary carnitine deficiency	Normal	*SLC25A1*	c.845G > C	p.R282P	Uncertain	Non-carrier of Primary carnitine deficiency
				*FTCD*	c.610C > T	p.R204W	Uncertain	

^a^By definition of the clinical diagnosis confirming the IEM (NB 1–8), all newly detected DNA variants were re-classified as pathogenic

**Fig. 3:**
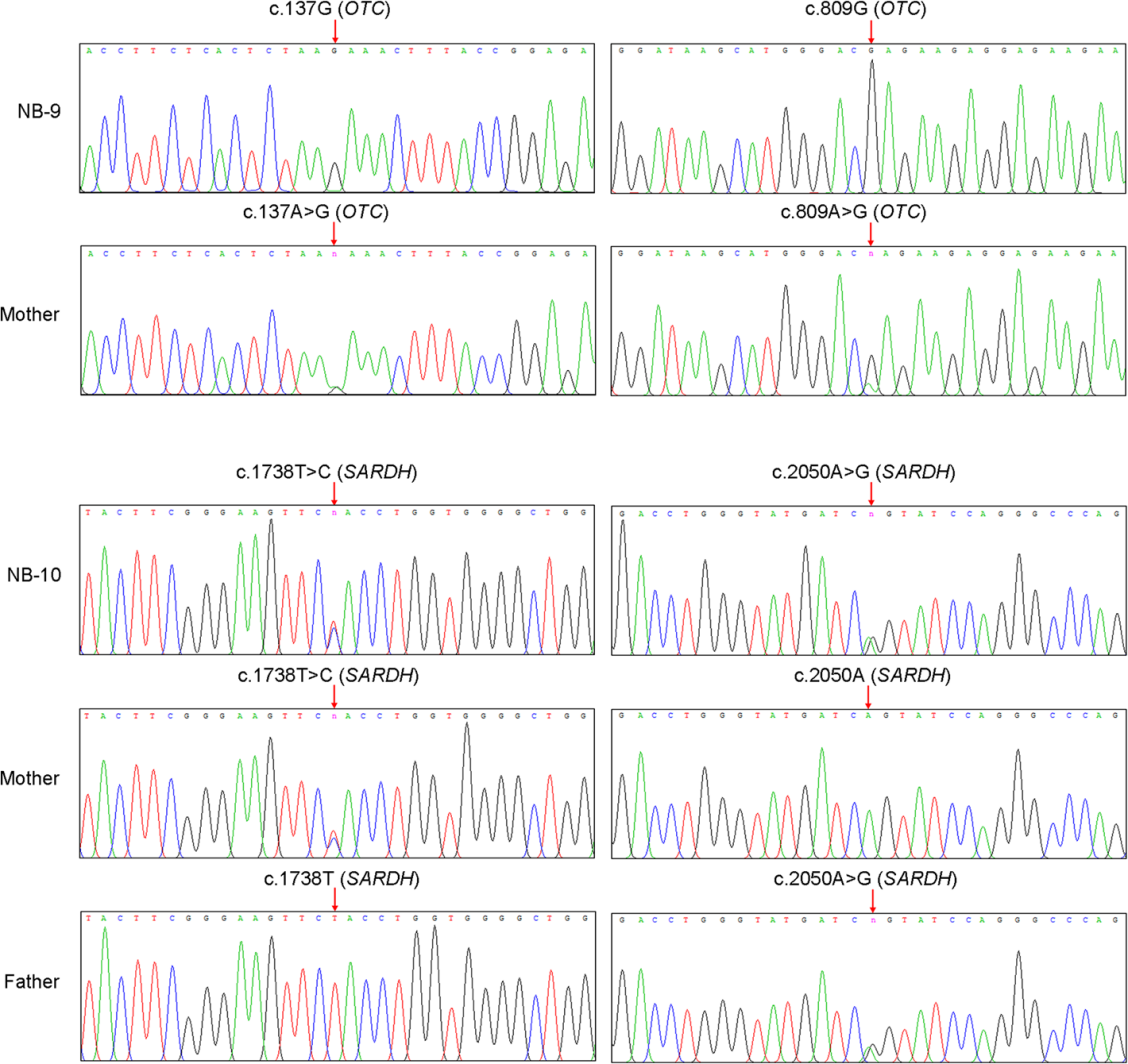
Confirmation of IEM DNA variants detected in newborns 9 and 10 with false positive MS MS results

In the false positive case of the newborn boy (NB-9) suspected with the X-linked disease ornithine transcarbamylase deficiency (OTC), we identified two missense DNA variants c.809A > G (p.Q270R, non-conservative amino acid change) and c.137A > G (p.K46R, conservative amino acid change) in his single copy *OTC* gene. Sanger sequencing subsequently confirmed both variants in the mother and the newborn boy, consistent with X-linked inheritance of OTC (Fig. 3). For newborn NB-10 with a suspected MS MS diagnosis of hyperalaninemia, we found no pathogenic variants associated with the causative pyruvate carboxylase deficiency gene *PC*. However, serendipitously, we identified compound heterozygous variants c.1738T > C (p.Y580H, non-conservative amino acid change) and c.2050A > G (p.S684G, non-conservative amino acid change) of uncertain significance in the *SARDH* gene (Fig. 3). Genomic DNA analysis of the family trio showed autosomal recessive inheritance in the proband for the individual parental carrier variants. In two other newborns NB-11 and NB-12 with a suspected diagnosis of maple syrup urine disease (Table 1), no DNA variants were detected in any of the three known causative genes *BCKDHA*, *BCKDHB* or *DBT*. However, in NB-11, compound heterozygous variants c.458G > A (p.R153Q, non-conservative amino acid change) and c.799C > T (p.R267W, non-conservative amino acid change) of uncertain significance were detected in the *MMACHC* gene associated with methylmalonic aciduria. Conversely, in NB-12, two compound heterozygous variants c.1823G > A (p.G608D, non-conservative amino acid change) and c.770A > G (p.Y257C, non-conservative amino acid change) of uncertain significance were detected in the *ETFDH* gene associated with glutaric aciduria type II.

**Fig. 4:**
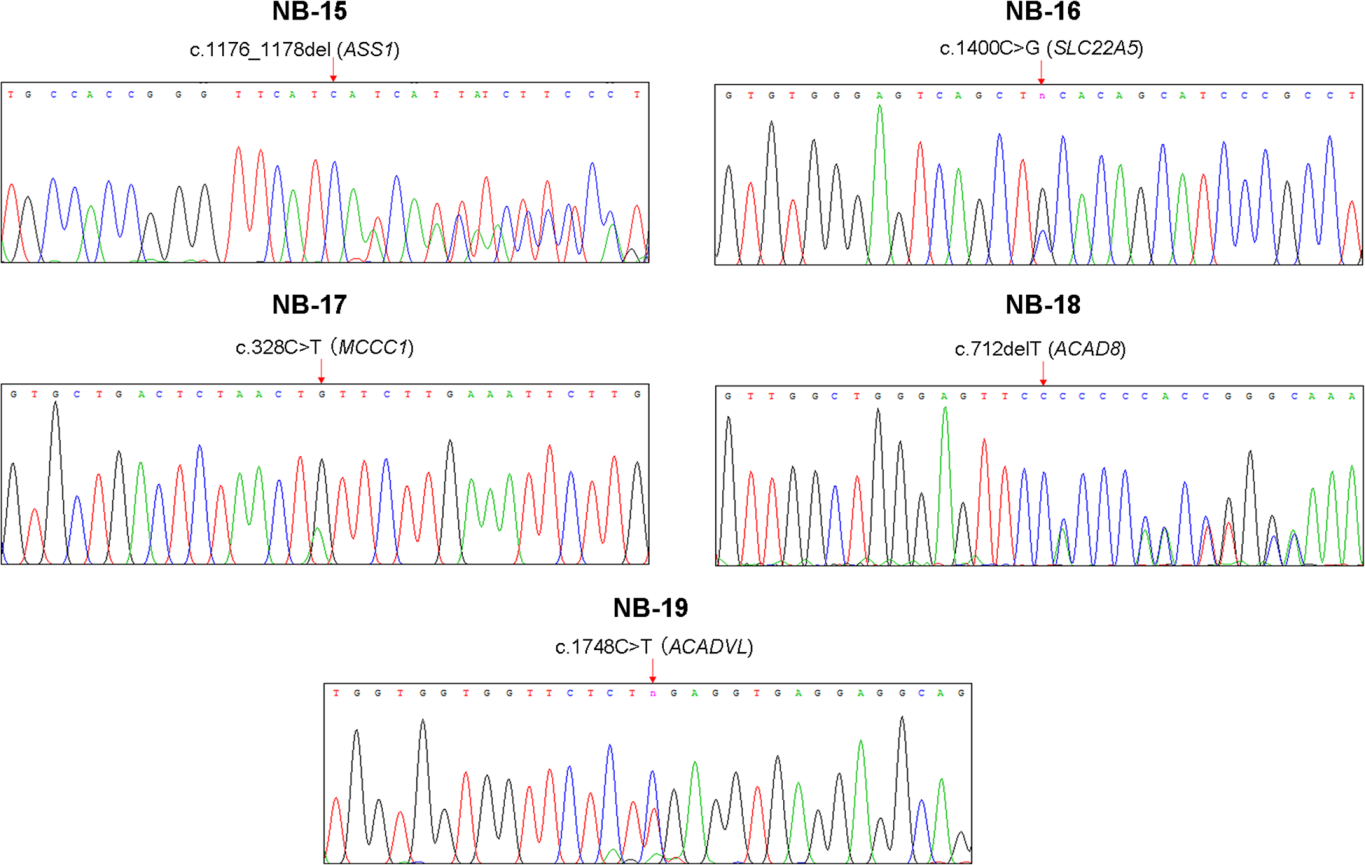
Sanger sequencing confirmation of IEM DNA variants detected in 5 newborns diagnosed with as normal (false positives)

In the 7 of the remaining 8 newborns (NB13–19) with false positive MS MS results for short chain acyl-CoA dehydrogenase deficiency (NB-13), citrullinemia type 1 (NB-14 and NB-15), primary carnitine deficiency (NB-16), 3 methylcrotonyl coenzyme A dehydrogenase deficiency (NB-17), isobutyryl-CoA dehydrogenase deficiency (NB-18) and very long-chain 3-OH acyl-CoA dehydrogenase deficiency (NB-19), we identified a single heterozygous variant of uncertain significance (carrier status) in the relevant genes associated with the indicated IEM. Sanger sequencing confirmed the variant carrier status predicted by the PEARS-101 assay in all of these 6 members (Fig. 4). For the exception NB-20 with suspected primary carnitine deficiency, no DNA variants were detected in the associated *SLC22A* gene. Follow up genotyping of NB13–20 by whole exome sequencing using alternative capture probes covering the exome regions of the relevant genes, gave concordant genotype results with the PEARS-101 assay.

## Discussion

In this pilot study, we developed a novel PCR exome sequencing based gene test termed PEARS-101 for detection of pathogenic variants associated with 101 different IEM and assessed the potential of this gene test for defining the mutations associated with the suspected IEM. Initial validation studies indicated that for each IEM gene, there was sufficient read coverage per exome probe to confidently call newborns with either homozygous or compound heterozygous mutation combinations. Preliminary studies of genomic DNA samples correctly identified the known IEM DNA variants, previously identified by Sanger sequencing. Further, in the newborn blood spots where the gene test defined DNA variants associated with various types of IEM, all variants were verified by standard Sanger sequencing. On this basis the PEARS-101 delivered high sensitivity and specificity for IEM mutation detection and correctly revealed genotypes that correlated with the final clinical diagnosis.

In application of the test to 9 newborns with clinically diagnosed with PKU, we showed, the bloodspot assay correctly diagnosed 8 (89%), finding compound heterozygote pathogenic variants. The one false negative involved a bloodspot detected with a single heterozygous *PAH* variant c.1197A > T (p.V399 V) which is known to be pathogenic [26]. The inability to identify a second pathogenic variant by the PEARS-101 assay was not due to detection failure of PCR since follow up whole exome sequencing using alternative gene probes confirmed the c.1197A > T variant and did not detect any other *PAH* gene variants. A plausible explanation is that the second mutation exists either in the 5 prime or 3 prime untranslated regions or deeper within intronic regions of the *PAH* gene that were not covered by the exome amplicons and possibly operates at the mRNA level, reducing expression of PAH enzyme levels. With patient consent, whole genome sequencing or cDNA analysis will be undertaken in an attempt to reveal the potential presence of a second pathogenic DNA variant in other regions of the *PAH* gene. In a prospective study of 211 bloodspots, the assay correctly identified the 2 true positives and the 209 true negative newborns, indicating a PPV and NPV of 100% for PKU.

Analysis of a small selected set of MS MS true positive (*n* = 8) and false positive (*n* = 12) bloodspots showed the ability of the PEARS-101 assay to assign a genotype that was consistent with the final IEM phenotype. For the true positives, the gene test identified compound heterozygote variants of uncertain significance for two cases of citrullinemia type 1, and one case each of methylmalonic acidemia, isobutyryl-CoA dehydrogenase deficiency, short chain acyl-CoA dehydrogenase deficiency and glutaric acid type II and heterozygous pathogenic variants in the two cases of methioninemia. Based on disease diagnosis by biochemical testing, these mutations were clearly pathogenic, and add to the known pathogenic variants causative of these IEM. In one false positive case (male NB-10) involving X-linked disease OTC, the test found two DNA variants in the *OTC* gene consistent with the original MS MS positive result. The variants identified predicted one conservative and one non-conservative amino acid change in cis, suggesting a potential disease causing defect in OTC enzyme activity. At birth, the MS MS metabolic profile bloodspot indicated a decreased ratio of level of citrulline, which is the primary metabolic defect in newborns with OTC. However, subsequent investigation and clinical examination of the boy at 10 months of age revealed normal blood citrulline levels and no obvious symptoms of neonatal OTC (such as episodic vomiting, lethargy, irritability, or failure to thrive). Of clinical significance, his mother has episodes of seizures, which is now believed to be associated with her *OTC* mutation carrier status. The parents have agreed to have their son constantly monitored, since symptoms of OTC are known to manifest after birth [27].

In regard to the remaining 11 MS MS false positives, the gene test was able to elucidate the basis of the discordance between positive MS MS results and negative biochemical testing results. While MS MS results indicated hyperalanineemia for NB-10 and maple syrup disease for NB-11 and NB-12, gene testing identified compound heterozygous variants of uncertain significance in other IEM genes *SARDH*, *MMACHC* and *ETFDH* related to sacrosinuria, methylmalonic aciduria and glutaric aciduria, respectively. For NB-10, the suspected IEM fell outside the scope of the metabolic diseases detectable by MS MS (Additional file 1: Table S1), providing an explanation for the discordant MS MS result. We believe the MS MS metabolic profiles indicating maple syrup disease for NB-11 and NB-12 may have been either erroneous or misinterpreted, and, further, did not show any evidence of metabolic disturbances indicative of methylmalonic aciduria and glutaric aciduria, respectively. From the analysis of 11 other false positives (NB 9–19), we found a correlation of heterozygous carrier status for variants in the genes relevant to each IEM suspected by MS MS. We suggest therefore that carrier gene status may be sufficient to reduce the threshold of functional enzyme activity, affecting the efficiency of the affected biochemical pathway, leading to an altered metabolic state that is detectable by MS MS, and thus accounting for the apparent false positive result. This is consistent with previous observations of a transient reduction in enzyme activity for some IEM in the early neonatal period [17].

In the newborn screening programs, the gold standard methodologies for first tier IEM screening are specific biochemical assays for common diseases like PKU and MS MS for around another 30 less common diseases. These tests are easy to perform, scalable and are relatively cheap, costing around $10 USD to analyse bloodspots [1] and 50–100 USD for family testing [18]. Overall, while the sensitivity of MS MS is very high there have been isolated reports of false negative MS MS results [16–18, 28]. Nonetheless, the specificity of MS MS is relatively low, with PPVs ranging from 15 to 25% [16–18, 29]. False positives can complicate clinical management of the newborn requiring unnecessary second tier testing that is associated with increased medical costs [17]. In addition, false positives can cause undue stress on anxious parents who are awaiting the final clinical diagnosis of their newborn child [30].

Our findings from this study, indicate that the PEARS-101 assay may have potential advantages as a second tier screening method to verify the primary biochemical testing results. Firstly, the gene test covers all relevant genes associated with 30 different IEM detectable by MS MS [17–21], allowing rapid analysis of the bloodspots for potentially pathogenic variants. Secondly, the test can provide a newborn genotype for 101 IEM and therefore obviates the need for follow up confirmatory Sanger or exome sequencing which adds significant cost. Thirdly, genotyping may help to identify and stratify positive MS MS results into true positive and false positives and so guide specific biochemical testing so that a rapid final diagnosis can be made. Reaching an early diagnosis for metabolic diseases is important for many IEM because early administration of treatment regimens or drugs can modulate disease symptoms and improve health outcomes [2]. Lastly, the knowledge of carrier DNA variants in both parents with potential pathogenicity for a specific IEM will also help guide reproductive options such prenatal diagnosis or preimplantation diagnosis for those couples who may decide to have another child.

Based on current multiplex PCR and sequencing costs, we estimate the cost of performing the PEARS-101 test is around $100 USD (trio testing $300 USD). In China, such a test used as a second tier screening method could attract a government subsidy to partly offset some costs to the patient, in cases where their newborn child is suspected to have an IEM by first-tier biochemical or MS MS testing. In this study, we also showed that every DNA variant detected by PEARS-101 assay was confirmed by Sanger sequencing. Next generation sequencing has an advantage over Sanger sequencing because it produces large numbers of single molecule allelic reads where the output is an alignment file (vcf) showing the sequencing reads of each exome, thus any true DNA variants will be repetitively represented in the profile, giving high confidence of the presence of a bonefide DNA variant. Any minor sequencing errors (false positives) will be easily identified by their lack of representation in the allelic sequencing reads. On this basis, we believe that exome based tests like PEARS-101 will eventually replace expensive Sanger sequencing, making Sanger sequencing largely redundant as either a diagnostic or a confirmatory methodology.

## Conclusions

Our findings demonstrate that gene testing is a useful method to help verify newborns positive for an IEM and guide specific biochemical confirmatory testing. In order to improve the current standard of care, we would advocate second tier gene testing for all newborns with a suspected IEM. By this approach, clinicians will be more empowered with informative genetic and biochemical results, allowing them to reach a definitive clinical diagnosis and, where appropriate, administer early treatments of the newborn to manage the disease symptoms over a lifetime.

## Additional files

Additional file 1: **Table S1.** Metabolic diseases and associated genes. The tabulated 101 metabolic diseases and their known causative genes were used to form the basis of the PEARS-101 test. Diseases highlighted in bold lettering comprise the 45 of 101 IEM detectable by MS MS. (TIF 5343 kb)

Additional file 2: **Figure S1.** QC parameters of the optimised PEARS-101 test. A. Agarose gel electrophoresis analysis of multiplex PCR products from P1 and P2 primer pools and the resulting library derived from mixing P1 and P2 products. B. Typical profile of sequencing depth for the PEARS-101 assay. C. Typical allelic ratios for the population of exome molecules derived and analyzed by the PEARS-101 assay. (TIF 4448 kb)

Additional file 3: **Figure S2.** Sanger sequencing confirmation of primary IEM mutations detected by PEARS-101 gene test in genomic DNA samples. (TIF 7581 kb)

Additional file 4: **Figure S3.** Sanger sequencing confirmation of secondary IEM mutations detected by PEARS-101 gene test in genomic DNA samples. (DOC 109 kb)

## Abbreviations

ACMG: American College of Medical Genetics
BWA: Burrows-Wheeler Aligner
CAH: Congenital adrenal hyperplasmia
CH: Congenital hypothyroidism
IEM: Inborn errors of metabolism
MS MS: Tandem mass spectroscopy
NGS: Next generation sequencing
NPV: Negative predictive value
OTC: Ornithine transcarbamylase deficiency
PAH: Phenylalanine hydroxylase
PE: Paired end
PEARS: PCR exome amplification and re-sequencing
PKU: Phenylketonuria
PPV: Positive predictive value
SNP: Single nucleotide polymorphism
WES: Whole exome sequencing
