# Supplementary figures and images for: A comprehensive multiplex PCR based exome-sequencing assay for rapid bloodspot confirmation of inborn errors of metabolism
Source: BMC Med Genet. 2019 Jan 6;20:3. doi: 10.1186/s12881-018-0731-5 (PMC6322297; doi:10.1186/s12881-018-0731-5)

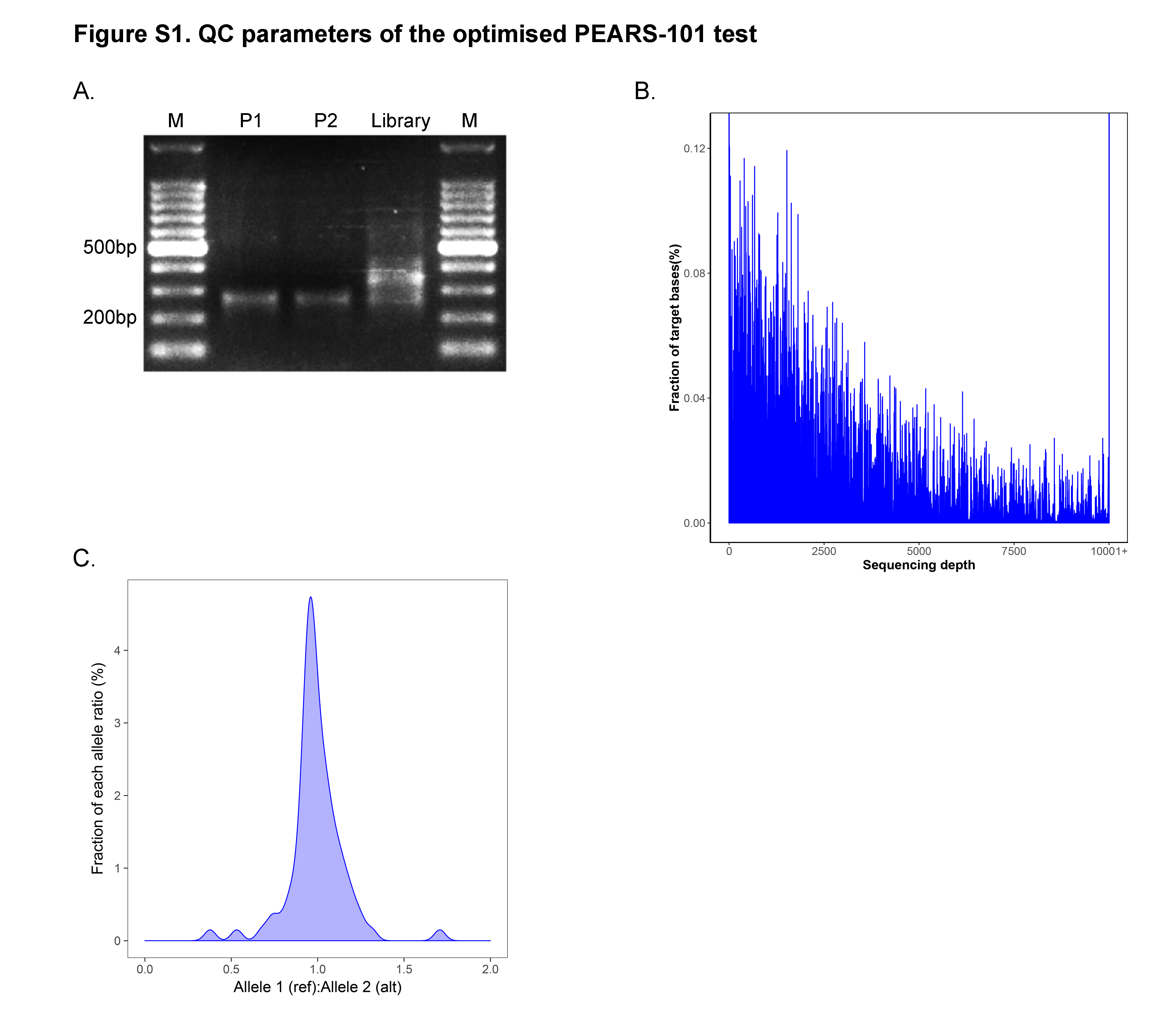

Supplement: Supplementary file 1 — Table S1. Metabolic diseases and associated genes. The tabulated 101 metabolic diseases and their known causative genes were used to form the basis of the PEARS-101 test. Diseases highlighted in bold lettering comprise the 45 of 101 IEM detectable by MS MS. (TIF 5343 kb) [file 12881_2018_731_MOESM1_ESM.tif]

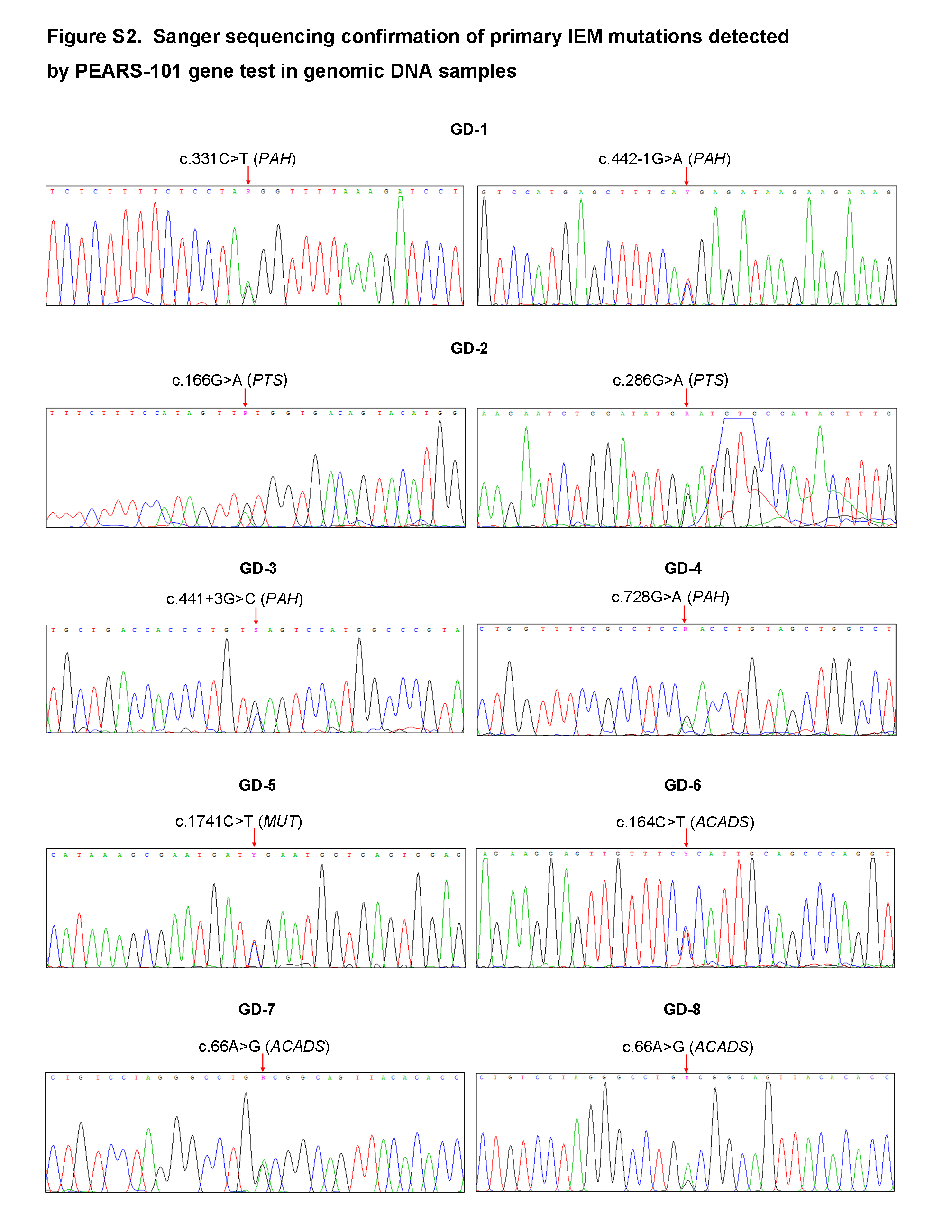

Supplement: Supplementary file 2 — Figure S1. QC parameters of the optimised PEARS-101 test. A. Agarose gel electrophoresis analysis of multiplex PCR products from P1 and P2 primer pools and the resulting library derived from mixing P1 and P2 products. B. Typical profile of sequencing depth for the PEARS-101 assay. C. Typical allelic ratios for the population of exome molecules derived and analyzed by the PEARS-101 assay. (TIF 4448 kb) [file 12881_2018_731_MOESM2_ESM.tif]

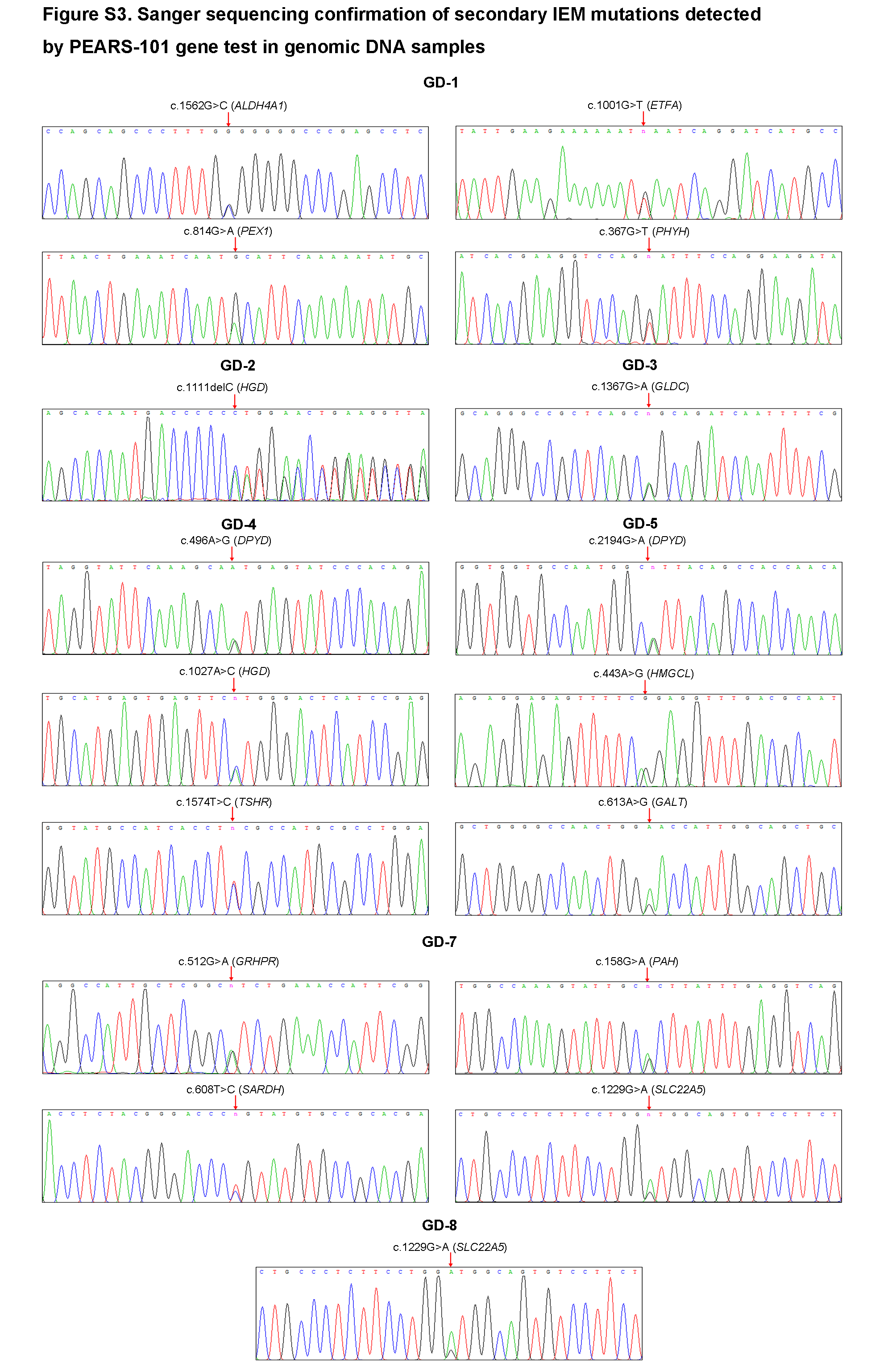

Supplement: Supplementary file 3 — Figure S2. Sanger sequencing confirmation of primary IEM mutations detected by PEARS-101 gene test in genomic DNA samples. (TIF 7581 kb) [file 12881_2018_731_MOESM3_ESM.tif]
