# Supplementary material for: A comprehensive multiplex PCR based exome-sequencing assay for rapid bloodspot confirmation of inborn errors of metabolism
Source: BMC Med Genet. 2019 Jan 6;20:3. doi: 10.1186/s12881-018-0731-5 (PMC6322297; doi:10.1186/s12881-018-0731-5)
Supplement: Supplementary file 4 — Figure S3. Sanger sequencing confirmation of secondary IEM mutations detected by PEARS-101 gene test in genomic DNA samples. (DOC 109 kb) [file 12881_2018_731_MOESM4_ESM.doc]

**Table S1:** **Metabolic diseases and causative genes**

| **Number** | **IEM (n=101)** | **Gene name (n = 122)** |
| --- | --- | --- |
| Amino Acidopathies and Organic Acidemia | | |
| 1 | **Propionic aciduria** | *PCCA, PCCB* |
| 2 | Holocarboxylase synthetase deficiency | *HLCS* |
| 3 | **Methylmalonic aciduria (Cbl A and Cbl B)** | *MMAB, MMADHC, MUT, MTRR, MMACHC* |
| 4 | **Methylmalonic aciduria (Cbl C and Cbl D)** | *MCEE* |
| 5 | **Methylmalonic aciduria** | *MMAA* |
| 6 | **Malonic aciduria** | *MLYCD* |
| 7 | **Isobutyryl-CoA dehydrogenase deficiency** | *ACAD8* |
| 8 | **2-methylbutyryl-CoA dehydrogenase deficiency** | *ACADSB* |
| 9 | **Beta-ketothiolase deficiency** | *ACAT1* |
| 10 | **Isovaleric aciduria** | *IVD* |
| 11 | **3-methylcrotonyl-CoA carboxylase deficiency** | *MCCC1, MCCC2* |
| 12 | **3-methylglutaconic aciduria** | *AUH* |
| 13 | Barth syndrome | *TAZ* |
| 14 | **3-hydroxy 3-methyl glutaric aciduria** | *HMGCL* |
| 15 | **Glutaric aciduria type I** | *GCDH* |
| 16 | **Glutaric aciduria type II** | *ETFA, ETFB, ETFDH* |
| 17 | Mevalonate kinase deficiency | *MVK* |
| 18 | **Phenylketonuria** | *PAH* |
| 19 | **Hyperphenylalaninemia**  **(tetrahydrobiopterin deficiency)** | *GCH1, PCBD1, PTS, QDPR* |
| 20 | **2-methyl 3-hydroxy butyric aciduria** | *HSD17B10* |
| 21 | **Tyrosinemia type I (hepatorenal tyrosinemia)** | *FAH* |
| 22 | **Tyrosinemia type II (oculocutaneous tyrosinemia)** | *TAT* |
| 23 | **Tyrosinemia type III**  **(4-hydroxyphenylpyruvate dioxygenase deficiency)** | *HPD* |
| 24 | Transient tyrosinemia of the newborn | *FAH, TAT, HPD* |
| 25 | **Maple syrup urine disease** | *BCKDHA, BCKDHB, DBT* |
| 26 | N-acetylglutamate synthase deficiency | *NAGS* |
| 27 | **Carbamylphosphate synthetase deficiency** | *CPS1* |
| 28 | **Ornithine transcarbamylase deficiency)** | *OTC* |
| 29 | **Citrullinemia type I**  **(arginosuccinate synthase deficiency)** | *ASS1* |
| 30 | **Citrullinemia type II (citrin deficiency)** | *SLC25A13* |
| 31 | **Argininosuccinic academia** | *ASL* |
| 32 | **Argininemia (arginase deficiency)** | *ARG1* |
| 33 | **hypermethioninemia (MAT I/III deficiency)** | *AHCY, GNMT, MAT1A* |
| 34 | **Homocystinuria (cystathionine beta-synthase deficiency)** | *CBS* |
| 35 | Alkaptonuria | *HGD* |
| 36 | Encephalopathy due to hydroxykynureninuria | *KYNU* |
| 37 | Hyperleucine-isoleucinemia | *BCAT1, BCAT2* |
| 38 | Dihydrolipoyl dehydrogenase (E3) deficiency | *DLD* |
| 39 | Beta-hydroxyisobutyryl CoA deacylase deficiency | *HIBCH* |
| 40 | Lysinuric protein intolerance | *SLC7A7* |
| 41 | Saccharopinuria | *AASS* |
| 42 | Cystathioninuria | *CTH* |
| 43 | **Hyperprolinemia type I** | *PRODH* |
| 44 | **Hyperprolinemia type II** | *ALDH4A1* |
| 45 | Hyper hydroxyprolinemia | *PRODH* |
| 46 | 2-hydroxyglutaric aciduria | *D2HGDH, L2HGDH, IDH2, SLC25A1* |
| 47 | Hawkinsinuria | *HPD* |
| 48 | Biotinidase deficiency | *BTD* |
| 49 | Fumarate hydratase deficiency | *FH* |
| 50 | **Hyperornithinemia-hyperammonemia-homocitrullinuria syndrome** | *SLC25A15* |
| Disorder of Sugar Metabolism | | |
| 51 | Classic galactosemia (galactosemia type I) | *GALT* |
| 52 | Galactokinase deficiency (galactosemia type II) | *GALK1* |
| 53 | Galactose epimerase deficiency (galactosemia type III) | *GALE* |
| 54 | D-glyceric aciduria | *GLYCTK* |
| 55 | Fructose-1, 6-diphosphatase deficiency | *FBP1* |
| 56 | Lactose intolerance | *LCT* |
| Disorders of Fatty Acid Metabolism | | |
| 57 | **Short-chain acyl-CoA dehydrogenasedeficiency** | *ACADS* |
| 58 | **Medium-chain acyl-CoA dehydrogenase deficiency** | *ACADM* |
| 59 | **Long-chain 3-OH acyl-CoA dehydrogenase deficiency** | *HADHA* |
| 60 | **Ethylmalonic encephalopathy** | *ETHE1* |
| 61 | Dicarboxylic aciduria | *SLC52A1* |
| 62 | **Mitochondrial trifunctional protein deficiency**  **(TFP deficiency)** | *HADHA, HADHB* |
| 63 | **Very long-chain 3-OH acyl-CoA dehydrogenase deficiency (VLCAD)** | *ACADVL* |
| 64 | **Primary carnitine deficiency** | *SLC22A5* |
| 65 | **Medium chain 3-ketoacyl-CoA thiolase deficiency** | *ACADM* |
| 66 | **Carnitine palmitoyltransferase deficiency I** | *CPT1A* |
| 67 | **Carnitine palmitoyltransferase deficiency II** | *CPT2* |
| 68 | **Carnitine-acylcarnitine translocase deficiency** | *SLC25A20* |
| 69 | **2,4-dienoyl-CoA reductase deficiency (De-Red)** | *NADK2* |
| 70 | Medium/short-chain L-3-hydroxyacyl-CoA dehydrogenase deficiency (M/SCHAD) | *HADH* |
| Peroxisomal Diseases | | |
| 71 | Zellweger syndrome  (severe form of Zellweger spectrum disorders) | *PEX1* |
| 72 | Neonatal adrenoleukodystrophy  (intermediate form of Zellweger spectrum disorders) | *PEX2* |
| 73 | Infantile refsum disease  (mildform of Zellweger spectrum disorders) | *PHYH, PEX7* |
| 74 | Zellweger-like syndrome (ZLS) | *HSD17B4* |
| 75 | Primary hyperoxaluria | *AGXT, GRHPR* |
| Disorders of Purine, Pyrimidine metabolism | | |
| 76 | Adenosine deaminase deficiency | *ADA* |
| 77 | Lesch-Nyhan syndrome | *HPRT1* |
| 78 | Kelley-Seegmiller syndrome | *HPRT1* |
| 79 | Adenine phosphoribosyltransferase deficiency | *APRT* |
| 80 | Orotic aciduria | *UMPS* |
| 81 | Dihydropyrimidine dehydrogenase deficiency | *DPYD* |
| 82 | Dihydropyrimidinase deficiency | *DPYS* |
| 83 | Beta-ureidopropionase deficiency | *UPB1* |
| Lactic acidemia, Hyperpyruvic metabolism | | |
| 84 | Pyruvate carboxylase deficiency | *PC* |
| 85 | Pyruvate dehydrogenase (E1) deficiency | *PDHA1* |
| 86 | Hyperglycinuria | *SLC6A20, SLC6A19, SLC36A2* |
| 87 | **Nonketotic hyperglycinemia** | *GLDC, AMT, GCSH* |
| 88 | Formiminoglutamic aciduria | *FTCD* |
| 89 | Valinemia (hypervalinemia/valine transaminase deficiency) | *BCAT1, BCAT2* |
| 90 | Histidinuria | *HAL* |
| Other IEM | | |
| 91 | Sarcosinuria | *SARDH* |
| 92 | Canavan disease | *ASPA* |
| 93 | Glutathione synthetase deficiency | *GSS* |
| 94 | Succinic semialdehyde dehydrogenase deficiency | *ALDH5A1* |
| 95 | Neonatal intrahepatic cholestasis caused by citrin deficiency (NICCD) | *ASS1, SLC25A13* |
| 96 | Glucose-6-phosphate dehydrogenase deficiency | *G6PD* |
| 97 | Congenital adrenal hyperplasia | *CYP11B1, CYP21A2* |
| 98 | Congenital hypothyroidism | *PAX8, TSHR, TSHB* |
| 99 | Pompe disease (acid maltase deficiency/glycogen storage disorder/glycogenosis II) | *GAA* |
| 100 | Fabry disease (alpha-galactosidase A deficiency) | *GLA* |
| 101 | Gyrate atrophy of the choroid and retina | *OAT* |

Diseases highlighted in bold lettering comprise the 45 IEM detectable by MS MS
